# Phylogeny and morphological analyses of *Penicillium* section *Sclerotiora* (Fungi) lead to the discovery of five new species

**DOI:** 10.1038/s41598-017-08697-1

**Published:** 2017-08-15

**Authors:** Xin-Cun Wang, Kai Chen, Zhao-Qing Zeng, Wen-Ying Zhuang

**Affiliations:** 1 State Key Laboratory of Mycology, Institute of Microbiology, Chinese Academy of Sciences, Beijing, 100101 China; 2 University of Chinese Academy of Sciences, Beijing, 100049 China

## Abstract

Phylogeny of *Penicillium* section *Sclerotiora* is still limitedly investigated. In this study, five new species of *Penicillium* are identified from the samples collected from different places of China, and named *P. austrosinicum*, *P. choerospondiatis*, *P. exsudans*, *P. sanshaense* and *P. verrucisporum*. The conidiophores of *P. austrosinicum* and *P. exsudans* are monoverticillate like most members of the section, while the rest species are biverticillate similar to the only two species *P. herquei* and *P. malachiteum* previously reported in the section *Sclerotiora*. The phylogenetic positions of the new taxa are determined based on the sequence data of ITS, *BenA*, *CaM* and *RPB2* regions, which reveals that all the species with biverticillate condiophores form a well-supported subclade in the section. The new *Penicillium* species clearly differ from the existing species of the genus in culture characteristics on four standard growth media, microscopic features, and sequence data. Morphological discrepancies are discussed between the new species and their allies.

## Introduction

*Penicillium* Link is one of the most common fungal genera occurring in diverse environments. They are able to decompose organic matters, and play an important role in biological circulation of biomasses in nature. They also display medicinal and industrial uses. Penicillin, the first antibiotic against gram-positive bacterial infections, has been produced by eight species in section *Chrysogena* ^1, 2^. A plethora of mycotoxins, such as citrinin, patulin and nephrotoxic ochratoxin A, are produced by different species^3^. The enzyme β-glucosidase, essential for biomass-based biofuel industry, is effectively secreted by *P. echinulatum* and *P. oxalicum* ^4, 5^. In food industry, *P. nalgiovense* and *P. salamii* are used as starter cultures for sausage fermentation^6^; *P. camemberti* for white cheese (Camembert and Brie) and *P. roqueforti* for blue cheese (roquefort, gorgonzola, stilton, gammelost, etc.)^7^. Apart from living as saprophytes, more than ten species are endophytes of diverse plants^8–10^.

*Penicillium* is lectotypified with one of the three originally species, *P. expansum*, by Pitt^11^. The genus is affiliated to the family Aspergillaceae, and contains two subgenera, *Aspergilloides* and *Penicillium*. It was further divided into 25 sections based on a four-gene phylogeny^12^. Recently, two new sections were established, while two other sections were synonymized as one^13^. In the past decades, significant advances have been achieved for the knowledge of species diversity of this group^11, 14–16^. More than 1000 *Penicillium* names were introduced in the past, and 354 species were generally accepted^17^. Fifty-seven additional species have recently been discovered^13, 18–27^.

The section *Sclerotiora* belonging to the subgenus *Aspergilloides* that contains 17 species was established by Houbraken and Samson^12^. Among them, *P. nodositatum* was excluded based on the results of sequence analyses; and *P. lilacinoechinulatum*, formerly considered as a synonym of *P. bilaiae*, was revived^28^. Seven more species were recently added including *P. alexiae*, *P. amaliae*, *P. arianeae*, *P. daejeonium*, *P. maximae*, *P. restingae* and *P. vanoranjei* ^28–30^. In China, four taxa (*P. adametzii*, *P. bilaiae*, *P. herquei* and *P. sclerotiorum*) of the section were recorded in the Flora Fungorum Sinicorum vol. 35 *Pennicilium* et Teleomorphi Cognati^31^. In this study, we describe five new species isolated from the soil and rotten fruit samples, which were collected in Guangdong, Hainan and Hunan provinces of China.

## Results

**Figure 1:**
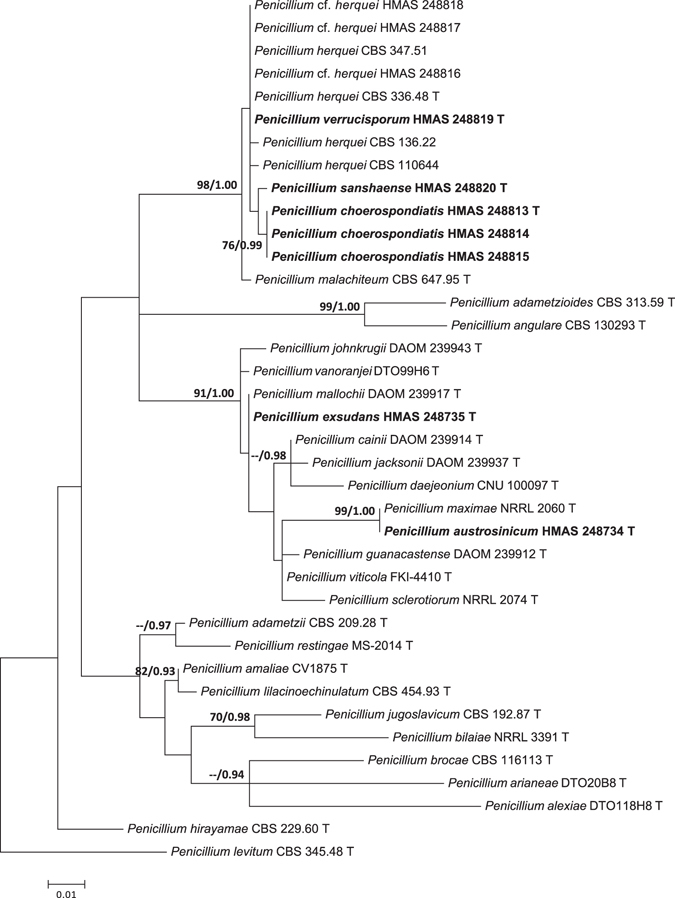
Maximum likelihood phylogenetic tree of *Penicillium* section *Sclerotiora* inferred from the sequences of the ITS region. Bootstrap values ≥70% (left) and posterior probability values ≥0.90 (right) are indicated at nodes.

The ITS data set contained 35 sequences of 521 bp. General Time Reversible (GTR) with gamma distribution (+G) was determined as the most suitable model for Maximum Likelihood (ML) analysis, and General Time Reversible with gamma distribution and invariant sites (GTR + I + G) was selected by Akaike Information Criterion as the best fit for Bayesian Inference (BI) analysis. In the ITS tree (Fig. 1), *P. choerospondiatis* and *P. sanshaense* were distinguished as sister groups that were associated with *P. herquei* and *P. verrucisporum*.

**Figure 2:**
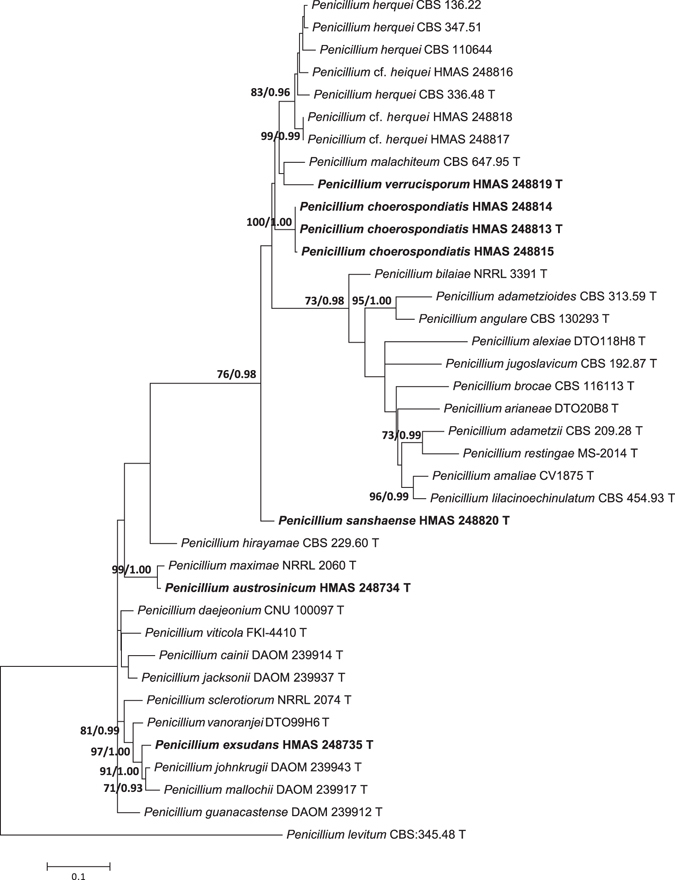
Maximum likelihood phylogenetic tree of *Penicillium* section *Sclerotiora* inferred from the sequences of the *BenA* region. Bootstrap values ≥70% (left) and posterior probability values ≥0.90 (right) are indicated at nodes.

**Figure 3:**
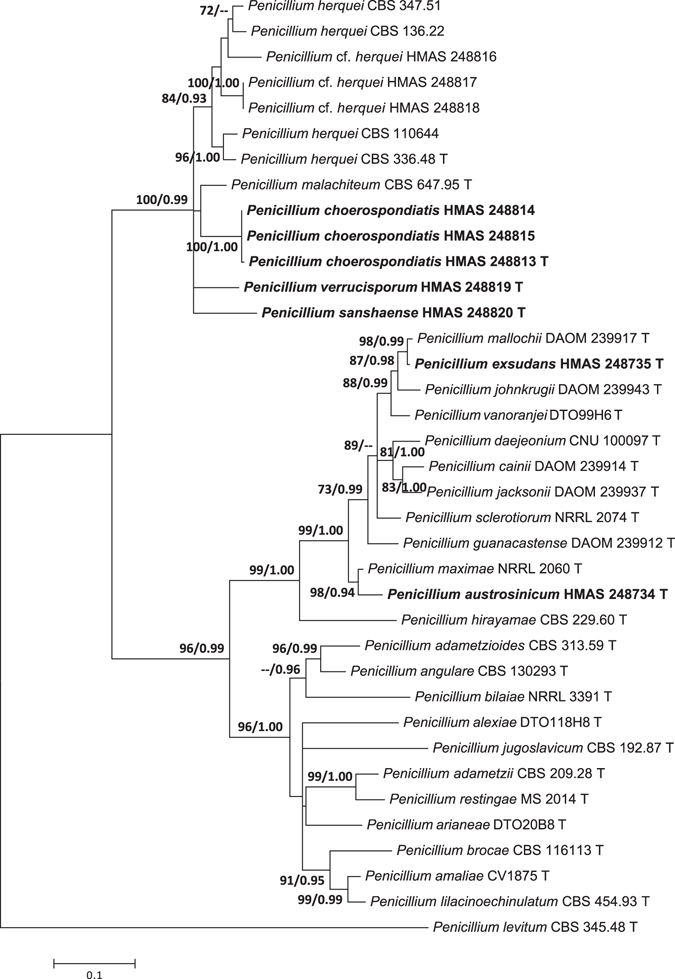
Maximum likelihood phylogenetic tree of *Penicillium* section *Sclerotiora* inferred from the sequences of the *CaM* region. Bootstrap values ≥70% (left) and posterior probability values ≥0.90 (right) are indicated at nodes.

**Figure 4:**
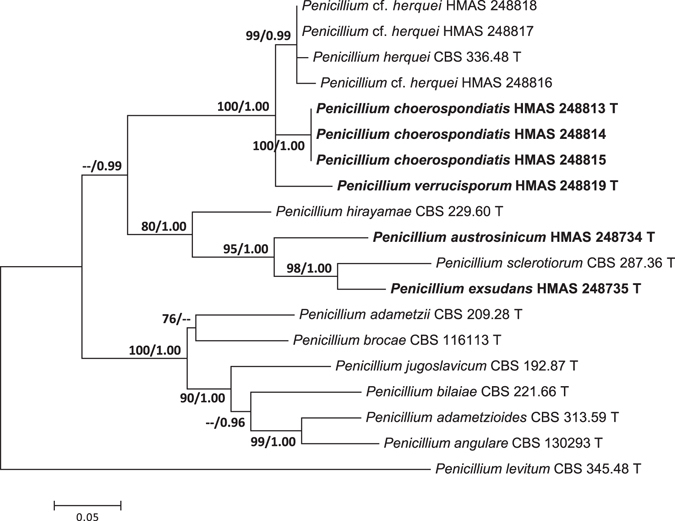
Maximum likelihood phylogenetic tree of *Penicillium* section *Sclerotiora* inferred from the sequences of the *RPB2* region. Bootstrap values ≥70% (left) and posterior probability values ≥0.90 (right) are indicated at nodes.

The other three single gene data sets included 35 sequences of 513 bp for *BenA* gene, 34 sequences of 592 bp for *CaM* partition, and 19 sequences of 1042 bp for the *RPB2*. The most suitable models for ML analyses were Kimura 2-parameter (K2) + G (*BenA*), Tamura-Nei (TN93) + I + G (*CaM*), and K2 + I + G (*RPB2*). The most suitable models for Bayesian Inference (BI) analyses were Tamura-Nei (TrN) + G (*BenA*), TrN + I + G (*CaM*), and equal-frequency Transition Model (TIMef) + I + G (RPB2). The individual gene analyses of the three genes supported the treatments of *P. austrosinicum*, *P. choerospondiatis*, *P. exsudans*, *P. sanshaense* and *P. verrucisporum* as valid new species (Figs 2–4).

**Figure 5:**
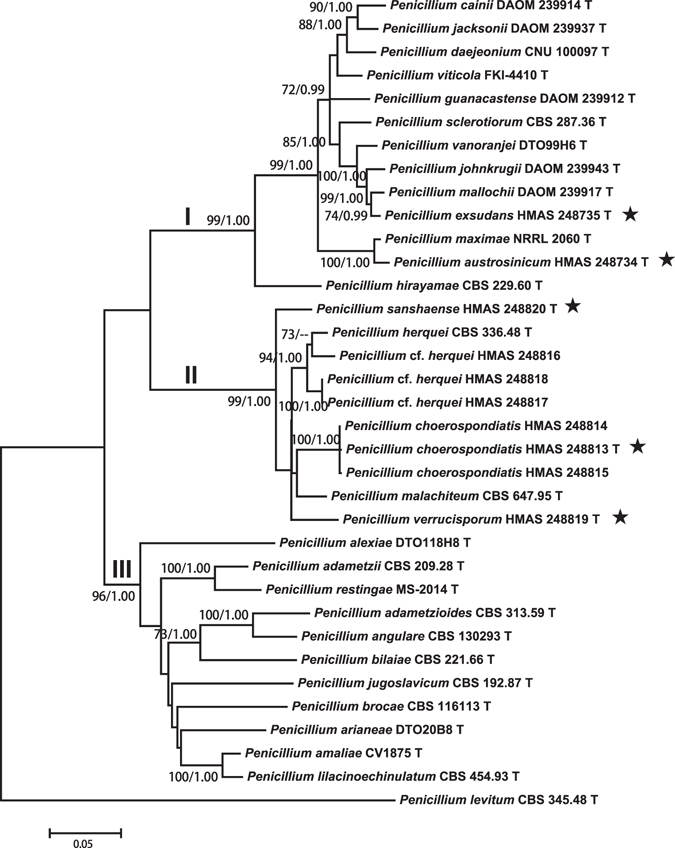
Maximum likelihood phylogenetic tree of *Penicillium* section *Sclerotiora* inferred from the concatenated sequences of ITS, *BenA*, *CaM* and *RPB2* regions. Bootstrap values ≥70% (left) and posterior probability values ≥0.90 (right) are indicated at nodes.

The combined data set consisted of 35 taxa with 2668 bp in length. The GTR + I + G model was determined as the most suitable model for ML and BI analyses. As shown in the four-gene phylogeny of *Penicillium* section *Sclerotiora* (Fig. 5), three subclades were recognized. Subclade I contained 13 species (MLBP/BIPP = 99%/1.00) including the newly described *P. austrosinicum* and *P. exsudans*. *Penicillium austrosinicum* was associated with *P. maximae* (MLBP/BIPP = 100%/1.00), and *P. exsudans* was a sister of *P. mallochii* (MLBP/BIPP = 74%/99%). The conidiophores of species in this subclade are all monoverticillate. Subclade II consisted of five species (MLBP/BIPP = 99%/1.00) of which three are newly established. The conidiophores of species in the subclade II are uniformly biverticillate. Subclade III accommodated the rest monoverticillate species in the section (MLBP/BIPP = 96%/1.00).

## Taxonomy

***Penicillium austrosinicum*** X.C. Wang & W.Y. Zhuang, sp. nov.

Figure 6

**Figure 6:**
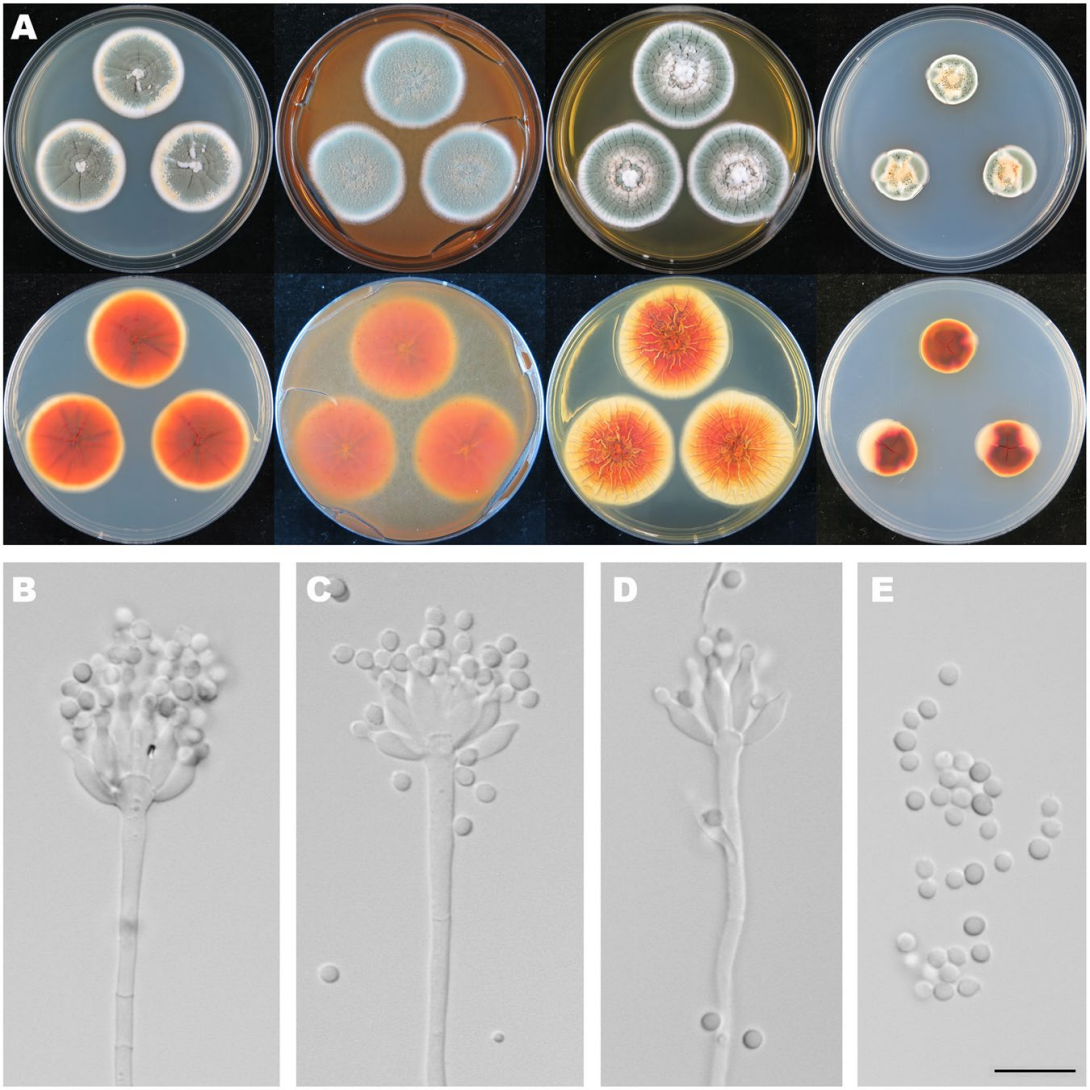
Colonial and microscopic morphology of *Penicillium austrosinicum* (HMAS 248734). (**A**) Colony phenotypes (top row left to right, obverse CYA, MEA, YES and CZ; bottom row left to right, reverse CYA, MEA, YES and CZ); (**B–D**) Conidiophores; (**E**) Conidia. Bars: E 10 µm, applies to B–D.

Fungal Names: FN570338

*Etymology*: The specific epithet refers to the locality of the type strain.

*DNA barcodes*: ITS KX885061, *BenA* KX885041, *CaM* KX885051, *RPB2* KX885032.

Colony diam, 7 d, 25 °C (unless stated otherwise): CYA 33–34 mm; CYA 30 °C 32–35 mm; CYA 37 °C no growth; CYA 5 °C no growth; MEA 35–37 mm; YES 37–40 mm; CZ 18–22 mm.

*Diagnosis*: *Penicillium austrosinicum* characterized by producing sclerotia on CYA at 25 °C, strictly monoverticillate conidiophores, ampulliform phialides, subglobose and rough-walled conidia.

On CYA 25 °C, 7 d: Colonies nearly circular, plain, conspicuously and radially sulcate, white mycelia appeared in centers, abundant cream to yellow sclerotia produced; margins moderately wide, entire; mycelia white; texture floccose; sporulation dense; conidia *en masse* dull green; soluble pigments absent; exudates clear and yellowish; reverse conspicuously and radially sulcate, orange in centers but buff at periphery. On MEA 25 °C, 7 d: Colonies nearly circular, plain, radially sulcate in central areas; margins wide, entire; mycelia white and orange; texture floccose; sporulation dense; conidia *en masse* greyish green; soluble pigments absent; exudates tiny and orange; reverse radially sulcate in central areas, orange in centers but yellow at periphery. On YES 25 °C, 7 d: Colonies nearly circular, protuberant in centers with white mycelia, radially and concentrically sulcate; margins moderately wide, entire; mycelia white; texture velutinous; sporulation dense; conidia *en masse* light green to green; soluble pigments absent; exudates absent; reverse conspicuously and radially sulcate, orange in centers but buff at periphery. On CZ 25 °C, 7 d: Colonies nearly circular or irregular, protuberant in centers with white and orange mycelia; margins narrow to moderately wide, entire or irregular; mycelia white; texture velutinous; sporulation moderately dense; conidia *en masse* greyish green; soluble pigments absent; exudates abundant, clear; reverse reddish brown in centers but orange and buff at periphery. Conidiophores strictly monoverticillate; stipes septate, smooth-walled, 35–175 × 2–3 µm, vesiculate; phialides ampulliform, 8–12, 6–9 × 2.5–3 µm; conidia subglobose, rough-walled, 2.7–3.3 × 2.5–3.3 µm.

*Typification*: CHINA. Guangdong Province, Shaoguan City, Shixing County, Chebaling National Nature Reserve, Xianrendong Village, 24°44′3″N 114°12′24″E, rotten fruit, 1 November 2015, Zhao-Qing Zeng, Xin-Cun Wang, Kai Chen & Yu-Bo Zhang, 10541 (holotype HMAS 248734, ex-type strain CGMCC 3.18410).

*Notes*: Fig. 1 shows that *P. austrosinicum* is the sister of *P. maximae* (MLBP/BIPP = 100%/1.00) in subclade I. Compared with the new species, *P. maximae* differs in sparse sporulation on CYA at 25 °C, orange mycelia on MEA at 25 °C, absence of sclerotia, the occasionally seen biverticillate conidiophores, ellipsoidal and smooth conidia, and faster growth rate on YES at 25 °C. The detailed morphological differences between *Penicillium austrosinicum* and related fungi are summarized in Table 1.

**Table 1:** Morphological comparison of the related *Penicillium* species.

	Conidiophore pattern	No. of metulae per verticil	Metula size (µm)	No. of phialides per metula	Phialide size (µm)	Conidial shape	Conidial walls	Conidial size (µm)	Diam on CYA at 25 °C after 7d (mm)	Diam on MEA at 25 °C after 7d (mm)	Diam on YES at 25 °C after 7d (mm)
*P. choerospondiatis*	biverticillate	3–5	10.5–15 × 3.5–4.5	5–8	9–12 × 3.3–4	ellipsoidal	finely roughened	4.5–6.5 × 3.3–4.5	11–12.5	26–29	16–19
*P. herquei*	biverticillate	4–6	10–12 × 3–5	6–10	7–10 × 2.5–3	ellipsoidal to apiculate	smooth to roughened	3.5–5 × 3–3.5	20–32	30–40	30–40
*P. malachiteum*	biverticillate	4–8	8–12 × 2.5–4	4–10	8–12 × 2–2.5	ellipsoidal	smooth to finely roughened	3–5 × 1.5–3.5	20–25	28–32	28–32
*P. sanshaense*	biverticillate	6–8	9–12 × 4–6.5	6–8	9–12 × 3–4	ellipsoidal	smooth	3–3.5 × 2–2.5	21–23	28–30	32–34
*P. verrucisporum*	biverticillate	5–8	9–10.5 × 3.5–5.5	5–8	7.5–8 × 2.5–3.5	ellipsoidal	roughened	3–3.5 × 2.5–3	25–27	36–37	43–44
*P. austrosinicum*	monoverticilate			8–12	6–9 × 2.5–3	subglobose	roughened	2.7–3.3 × 2.5–3.3	33–34	35–37	37–40
*P. maximae*	monoverticilate			8–16	6.5–10 × 2.5–3	ellipsoidal	smooth	3–3.5 × 2.5–3	34–37	34–37	40–43
*P. exsudans*	monoverticilate			6–12	7.5–10 × 3.3–4.5	subglobose to ellipsoidal	finely roughened	2.7–3.3 × 2.3–3	34–36	31–33	39–42
*P. johnkrugii*	monoverticilate			n.a.	7–11 × 2–3	globose to subglobose	finely roughened	2–3	30–38	26–36	28–38
*P. mallochii*	monoverticilate			n.a.	7–10 × 2–3	globose to subglobose	finely roughened	2.5–3.5 × 2–2.5	29–39	24–35	n.a.

n.a.: data not available.

***Penicillium choerospondiatis*** X.C. Wang & W.Y. Zhuang, sp. nov.

Figure 7

**Figure 7:**
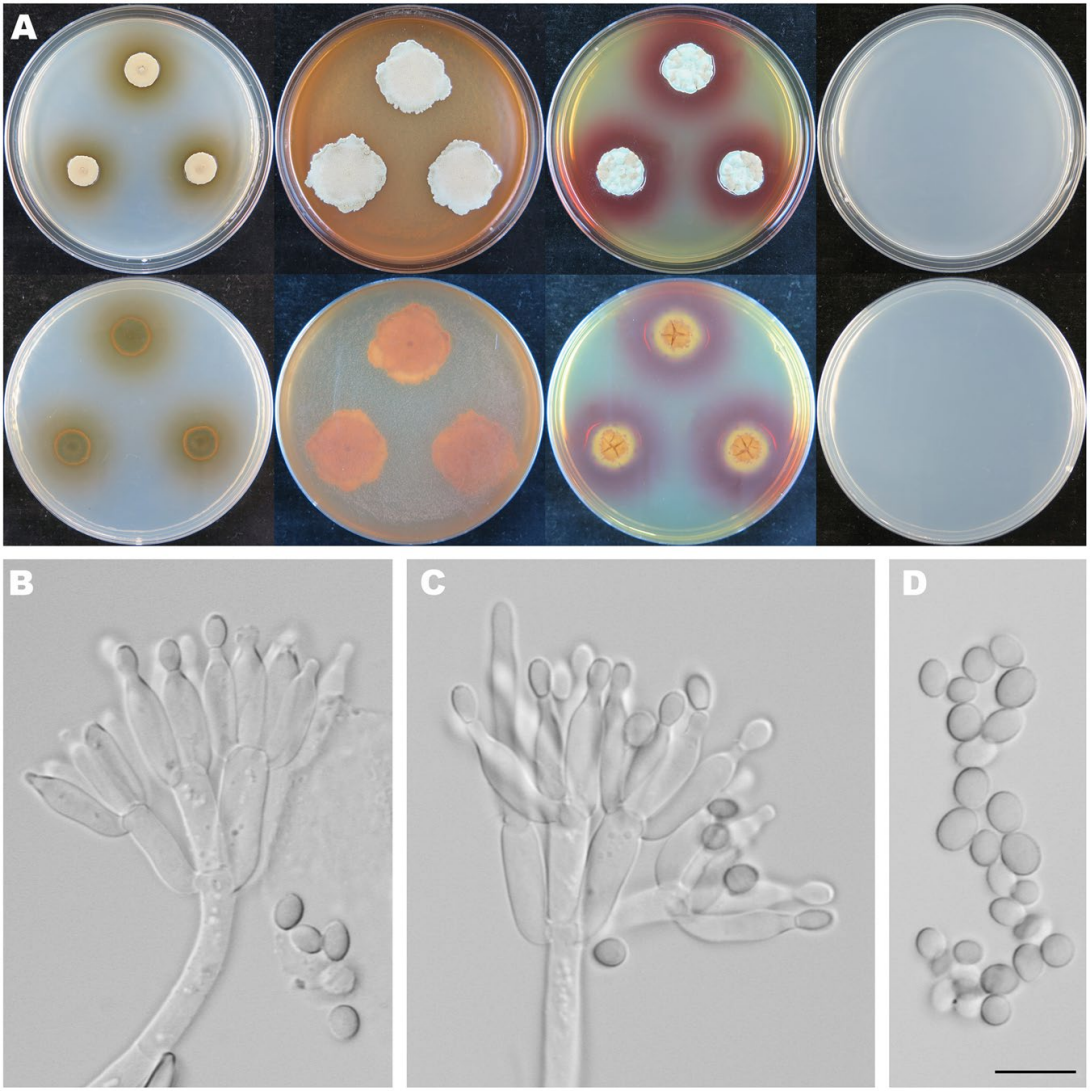
Colonial and microscopic morphology of *Penicillium choerospondiatis* (HMAS 248813). (**A**) Colony phenotypes (top row left to right, obverse CYA, MEA, YES and CZ; bottom row left to right, reverse CYA, MEA, YES and CZ); (**B–C**) Conidiophores; (**D**) Conidia. Bars: D 10 µm, applies to B–C.

Fungal Names: FN570333

*Etymology*: The specific epithet refers to the host plant *Choerospondias axillaris*.

*DNA barcodes*: ITS KX885063, *BenA* KX885043, *CaM* KX885053, *RPB2* KX885034.

Colony diam, 7 d, 25 °C (unless stated otherwise): CYA 11–12.5 mm; CYA 37 °C no growth; CYA 5 °C no growth; MEA 26–29 mm; YES 16–19 mm; CZ nogrowth.

*Diagnosis*: *Penicillium choerospondiatis* characterized by crustose colonies on CYA, MEA and YES at 25 °C, no growth on CZ at 25 °C, short and smooth stipes, biverticillate conidiophores, ampulliform phialides, large, ellipsoid and finely roughened conidia, and occurring on fruits of *Choerospondias axillaris*.

On CYA 25 °C, 7 d: Colonies nearly circular, convex, crustose, conidia dislodged onto the cover when the plates inverted; margins narrow, entire or undulate; mycelia white; texture velutinous; sporulation very dense; conidia *en masse* greyish green; soluble pigments light brown; exudates absent; reverse nearly black but yellowish brown at margins. On MEA 25 °C, 7 d: Colonies irregular, plain, crustose, abundant conidia dislodged onto the cover when the plates inverted; margins irregular; mycelia white; texture velutinous; sporulation very dense; conidia *en masse* dull green; soluble pigments absent; exudates absent; reverse light brown to brown. On YES 25 °C, 7 d: Colonies nearly circular or irregular, concave in centers, irregularly sulcate, crustose, abundant conidia dislodged onto the cover when the plates inverted; margins irregular; mycelia white; texture velutinous; sporulation very dense; conidia *en masse* dull green and yellowish brown; soluble pigments abundant, reddish brown; exudates absent; reverse rimose in centers, brown and yellowish brown, but nearly black at periphery. On CZ 25 °C, 7 d: No growth. Conidiophores biverticillate, a minor proportion monoverticillate and terverticillate; stipes septate, smooth-walled, 100–135 × 4–6 µm; metulae cylindrical, 3–5, 10.5–15 × 3.5–4.5 µm; phialides ampulliform, 5–8 per metula, 9–12 × 3.3–4 µm; conidia ellipsoidal, finely rough-walled, 4.5–6.5 × 3.3–4.5 µm.

*Typification*: CHINA. Hunan Province, Hengyang City, Hengyang County, Goulou town, Goulou Mountain National Forest Park, 27°06′42″N 112°36′28″E, on fruits of *Choerospondias axillaris*, 24 October 2015, Zhao-Qing Zeng, Xin-Cun Wang, Kai Chen & Yu-Bo Zhang, strain, Xin-Cun Wang, XCW_SN001 (holotype HMAS 248813, ex-type strain CGMCC 3.18411).

*Other specimens examined*: CHINA. Guangdong Province, Shaoguan City, Shixing County, Chebaling National Nature Reserve, 24°43′41″N 114°15′22″E, on fruits of *C. axillaris*, 31 October 2015, Zhao-Qing Zeng, Xin-Cun Wang, Kai Chen & Yu-Bo Zhang, strain, Xin-Cun Wang, XCW_SN002 (HMAS 248814); Songshukeng Village, 24°43′13″N 114°16′15″E, on fruits of *C. axillaris*, 2 November 2015, Zhao-Qing Zeng, Xin-Cun Wang, Kai Chen & Yu-Bo Zhang, strain, Xin-Cun Wang, XCW_SN049 (HMAS 248815).

*Notes*: *Penicillium herquei* and *P. malachiteum* were the only known taxa producing biverticillate conidiophores in section *Sclerotiora*. When the three new species with the same type of conidiophores are added, they turn out to be all together forming a well-supported subclade (Fig. 1, MLBP/BIPP = 99%/1.00).

As sister of the new species (Fig. 1), *P. malachiteum*, originally isolated from soil in Japan^32^, has longer and verrucose stipes (200–320 vs 100–135), more metulae per verticil (4–8 vs 3–5), smaller metulae and conidia, and faster growth rate on CYA and YES at 25 °C^28^. *Penicillium herquei* is also similar in colonial morphology, but differs in longer and roughened stipes (200–400 vs 100–135), smaller metulae, phialides and conidia, and growing faster on CYA, MEA and YES at 25 °C^11,28^. The morphological differences among the related taxa are summarized in Table 1.

Although the three strains of *P. choerospondiatis* were from different sites, they are morphologically identical. As to their sequence divergences, very few variations were detected. Their ITS and *RPB*2 regions are basically the same, six bp differences are found in the *BenA* gene, and three bp divergences exist in the *CaM* region among collections, which are treated as infraspecific variations.

***Penicillium exsudans*** X.C. Wang & W.Y. Zhuang, sp. nov.

Figure 8

**Figure 8:**
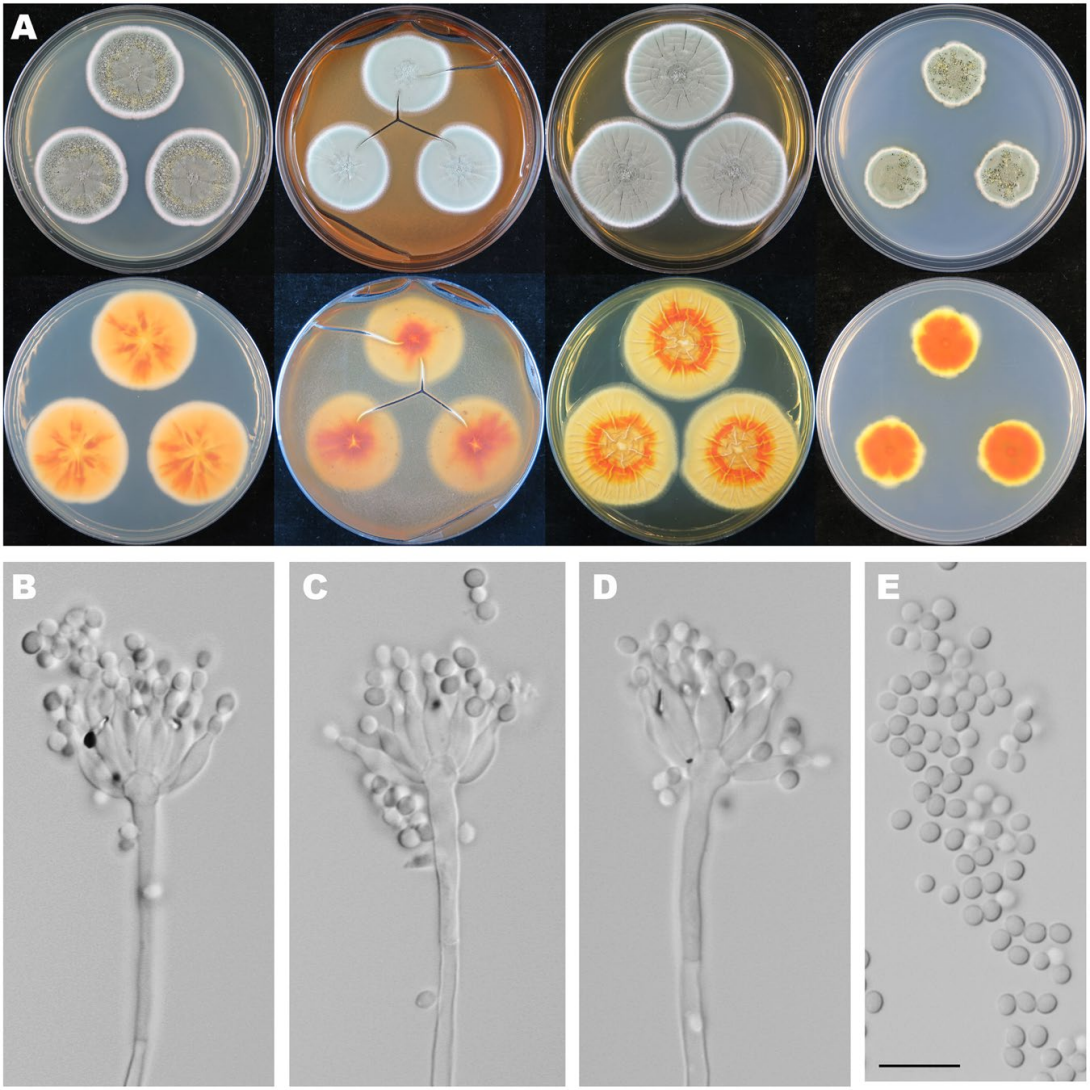
Colonial and microscopic morphology of *Penicillium exsudans* (HMAS 248735). (**A**) Colony phenotypes (top row left to right, obverse CYA, MEA, YES and CZ; bottom row left to right, reverse CYA, MEA, YES and CZ); (**B–D**) Conidiophores; (**E**) Conidia. Bars: E 10 µm, applies to B–D.

Fungal Names: FN570336

*Etymology*: The specific epithet refers to the abundant exudates produced on CYA and CZ at 25 °C.

*DNA barcodes*: ITS KX885062, *BenA* KX885042, *CaM* KX885052, *RPB2* KX885033.

Colony diam, 7 d, 25 °C (unless stated otherwise): CYA 34–36 mm; CYA 37 °C no growth; CYA 5 °C no growth; MEA 31–33 mm; YES 39–42 mm; CZ 22–25 mm.

*Diagnosis*: *Penicillium exsudans* characterized by strictly monoverticillate conidiophores, ampulliform phialides, subglobose to ellipsoidal and finely roughened conidia.

On CYA 25 °C, 7 d: Colonies nearly circular, plain, conspicuously and radially sulcate in central areas; margins entire; mycelia white; texture floccose; sporulation dense; conidia *en masse* green to greyish green; soluble pigments absent; exudates abundant, yellow near centers and clear at periphery; reverse conspicuously and radially sulcate in central areas, orange and bright yellow in centers but white at periphery. On MEA 25 °C, 7 d: Colonies nearly circular, plain, radially sulcate in central areas; margins moderately wide, entire; mycelia white; texture velutinous; sporulation dense; conidia *en masse* greyish green; soluble pigments absent; exudates absent; reverse radially sulcate in central areas, brownish red in centers but buff at periphery. On YES 25 °C, 7 d: Colonies nearly circular, plain, conspicuously and radially sulcate; margins wide, up to 2 mm, entire; mycelia white; texture velutinous; sporulation dense; conidia *en masse* greyish green; soluble pigments absent; exudates absent; reverse orange in centers but buff at periphery. On CZ 25 °C, 7 d: Colonies nearly circular or irregular, protuberant in centers; margins narrow to moderately wide, irregular; mycelia white; texture velutinous; sporulation dense; conidia *en masse* bluish green to greyish green; soluble pigments absent; exudates abundant, yellow or clear; reverse orange in centers but yellow to buff at periphery. Conidiophores strictly monoverticillate; stipes septate, smooth-walled, 60–130 × 2.5–4 µm, vesiculate or not; phialides ampulliform, 6–12, 7.5–10 × 3.3–4.5 µm; conidia subglobose to ellipsoidal, finely rough-walled, 2.7–3.3 × 2.3–3 µm.

*Typification*: CHINA. Guangdong Province, Shaoguan City, Shixing County, Chebaling National Nature Reserve, Xianrendong Village, 24°44′3″N 114°12′24″E, rotten fruit, 1 November 2015, Zhao-Qing Zeng, Xin-Cun Wang, Kai Chen & Yu-Bo Zhang, strain, Xin-Cun Wang, XCW_SN071 (holotype HMAS 248735, ex-type strain CGMCC 3.18412).

*Notes*: In Fig. 1, *P. exsudans* clustered with *P. mallochii* and *P. johnkrugii* (MLBP/BIPP = 99%/1.00) in subclade I. Compared with the new species, *P. mallochii* has yellow mycelia and crustose colonies on MEA at 25 °C, and longer stipes (53–380 vs 60–130)^33^. *Penicillium johnkrugii* differs in producing sclerotia on CYA, MEA and YES at 25 °C, not producing conidia on CYA at 25 °C, longer stipes (85–230 vs 60–130), and slower growth on YES at 25 °C^34^.

***Penicillium sanshaense*** X.C. Wang & W.Y. Zhuang, sp. nov.

Figure 9

**Figure 9:**
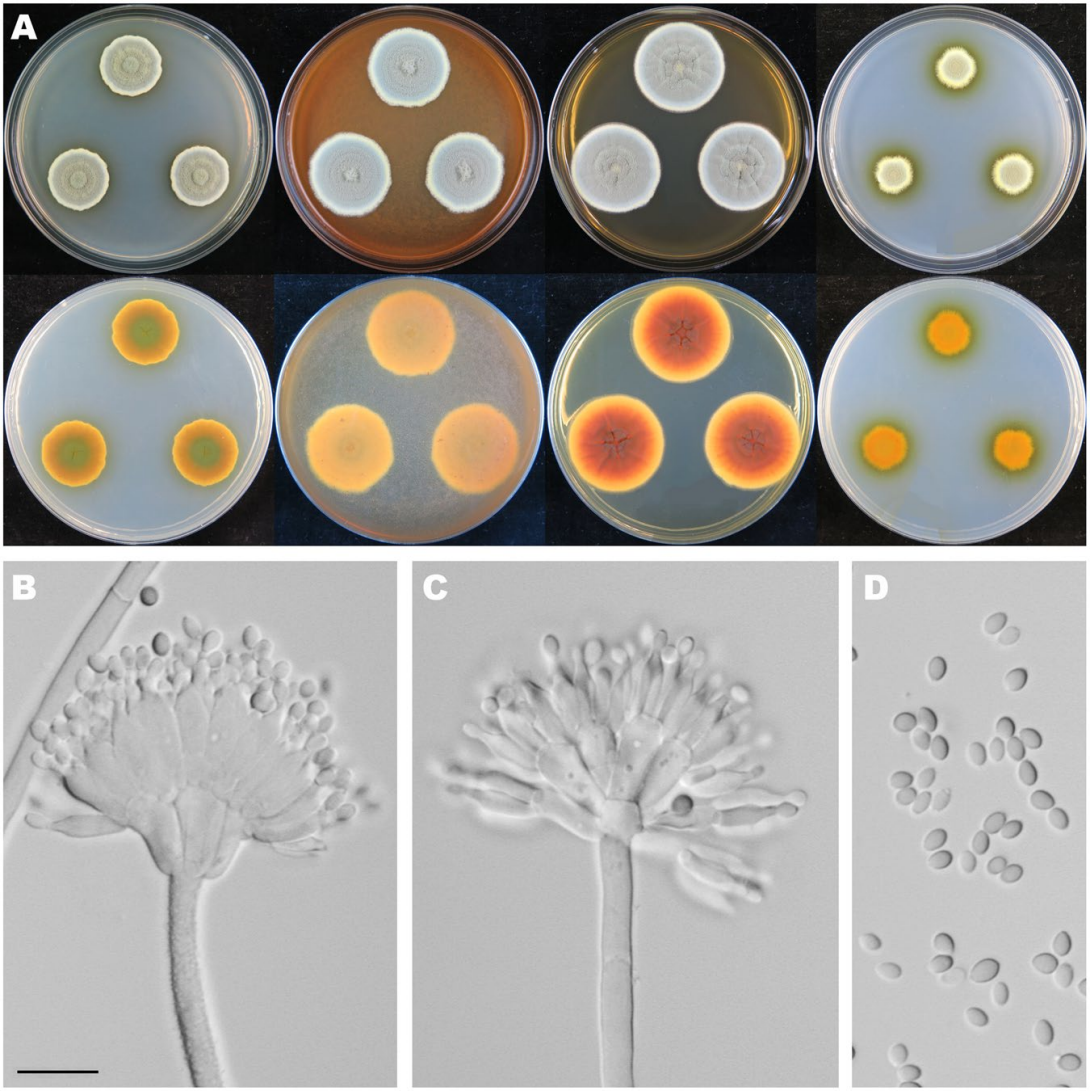
Colonial and microscopic morphology of *Penicillium sanshaense* (HMAS 248820). (**A**) Colony phenotypes (top row left to right, obverse CYA, MEA, YES and CZ; bottom row left to right, reverse CYA, MEA, YES and CZ); (**B–C**) Conidiophores; (**D**) Conidia. Bars: B 10 µm, applies to C–D.

Fungal Names: FN570337

*Etymology*: The specific epithet refers to the locality of the type strain.

*DNA barcodes*: ITS KX885070, *BenA* KX885050, *CaM* KX885060, *RPB2* n.a.

Colony diam, 7 d, 25 °C (unless stated otherwise): CYA 21–23 mm; CYA 37 °C no growth; CYA 5 °C no growth; MEA 28–30 mm; YES 32–34 mm; CZ 14–16 mm.

*Diagnosis*: *Penicillium sanshaense* characterized by long stipes, biverticillate conidiophores, ampulliform phialides, ellipsoidal and smooth conidia.

On CYA 25 °C, 7 d: Colonies nearly circular, protuberant in centers, concentrically sulcate; margins undulate; mycelia yellow; texture floccose; sporulation dense; conidia *en masse* greyish green; soluble pigments light greenish yellow; exudates clear or absent; reverse rimose and brown in centers but yellow to yellowish brown at periphery. On MEA 25 °C, 7 d: Colonies nearly circular, plain, funiculate at centers, concentrically sulcate; margins undulate, hairy; mycelia yellow; texture floccose; sporulation dense; conidia *en masse* greyish green; soluble pigments absent; exudates absent; reverse orange in central areas but yellowish white at periphery. On YES 25 °C, 7 d: Colonies nearly circular, concave in centers, concentrically and radially sulcate; margins moderately wide, entire; mycelia yellow; texture velutinous; sporulation dense; conidia *en masse* greyish green; soluble pigments absent; exudates absent; reverse radially sulcate, reddish brown in centers but yellow to orange at periphery. On CZ 25 °C, 7 d: Colonies nearly circular or irregular, convex, greenish yellow at centers; margins hairy; mycelia yellow; texture floccose; sporulation dense; sporulation dense; conidia *en masse* greyish green; soluble pigments light brown; exudates absent; reverse light yellowish brown in centers, brown in central areas, but yellow at periphery. Conidiophores biverticillate, a minor proportion terverticillate; stipes septate, smooth- to rough-walled, 200–500 × 3–4 µm; metulae 6–8, slightly swollen at the apices, 9–12 × 4–6.5 µm; phialides ampulliform, tapering into very thin neck, 6–8 per metula, 9–12 × 3–4 µm; conidia ellipsoidal, smooth-walled, 3–3.5 × 2–2.5 µm.

*Typification*: CHINA. Hainan Province, Sansha City, Xisha Islands, Yongxing Island, 16°50′41″N 112°20′50″E, soil, 29 March 2015, Ye-Wei Xia, strain, Kai Chen ZC97 (holotype HMAS 248820, ex-type strain CGMCC 3.18413).

*Notes*: Phylogenetically, *P. sanshaense* represents an independent lineage in subclade II (Fig. 1). *Penicillium* cf. *herquei* (HMAS 248817, HMAS 248818) is similar in smooth conidia, but differs in less metulae per verticil (2–5 vs 6–8), longer metulae obviously swollen at the apices, shorter phialides, and faster growth on CYA, MEA and YES at 25 °C.

***Penicillium verrucisporum*** X.C. Wang & W.Y. Zhuang, sp. nov.

Figure 10

**Figure 10:**
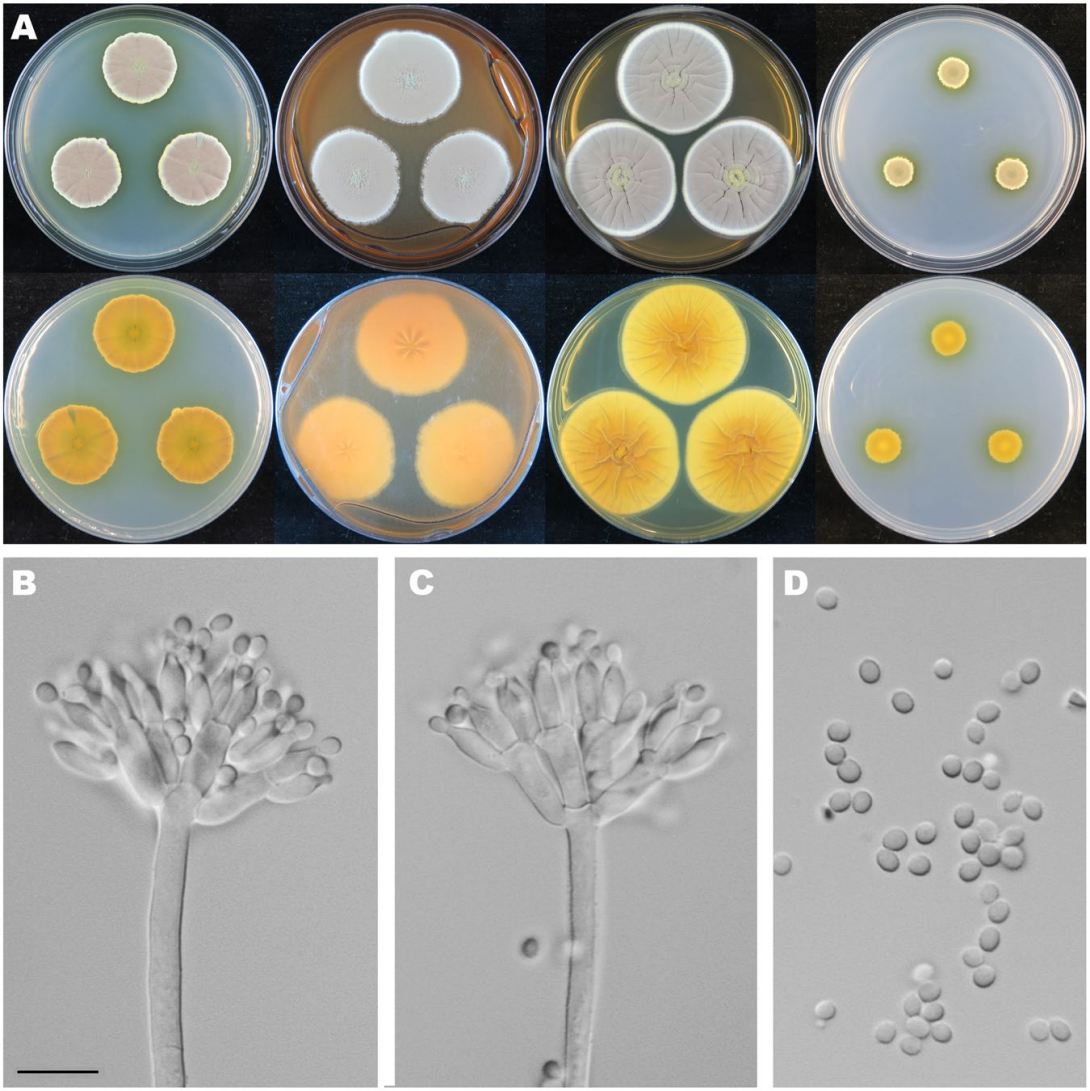
Colonial and microscopic morphology of *Penicillium verrucisporum* (HMAS 248819). (**A**) Colony phenotypes (top row left to right, obverse CYA, MEA, YES and CZ; bottom row left to right, reverse CYA, MEA, YES and CZ); (**B–C**) Conidiophores; (**D**) Conidia. Bars: B 10 µm, applies to C–D.

Fungal Names: FN570339

*Etymology*: The specific epithet refers to the warted walls of conidia.

*DNA barcodes*: ITS KX885069, *BenA* KX885049, *CaM* KX885059, *RPB2* KX885040.

Colony diam, 7 d, 25 °C (unless stated otherwise): CYA 25–27 mm; CYA 37 °C no growth; CYA 5 °C no growth; MEA 36–37 mm; YES 43–44 mm; CZ 11–12.5 mm.

*Diagnosis*: *Penicillium verrucisporum* characterized by comparatively short stipes, biverticillate conidiophores, ampulliform phialides, ellipsoidal and roughened conidia.

On CYA 25 °C, 7 d: Colonies nearly circular, plain, conspicuously and radially sulcate; margins narrow, undulate; mycelia yellow; texture velutinous; sporulation dense; conidia *en masse* greyish green to dull purplish brown; soluble pigments greenish yellow; exudates absent; reverse radially sulcate, greenish brown in centers but yellowish brown at periphery. On MEA 25 °C, 7 d: Colonies oblong, plain, funiculate in centers, radially sulcate in central areas or not; margins narrow to moderately wide, entire; mycelia white; texture velutinous; sporulation dense; conidia *en masse* dull greyish green; soluble pigments absent; exudates absent; reverse radially sulcate in central areas, yellowish to orangish brown in centers but buff at periphery. On YES 25 °C, 7 d: Colonies nearly circular, radially and concentrically sulcate; margins moderately wide, entire; mycelia yellow; texture velutinous; sporulation dense; conidia *en masse* greyish green; soluble pigments absent; exudates absent; reverse radially and concentrically sulcate, orangish brown in centers but buff at periphery. On CZ 25 °C, 7 d: Colonies nearly circular, convex; margins narrow, irregular; mycelia yellow; texture velutinous; sporulation dense; conidia *en masse* greyish green; soluble pigments greenish yellow; exudates absent; reverse buff in centers but yellowish brown at periphery. Conidiophores biverticillate; stipes septate, finely rough-walled, 125–200 × 3.5–4 µm; metulae 5–8, slightly swollen at the apices or not, 9–10.5 × 3.5–5.5 µm; phialides ampulliform, 5–8 per metula, 7.5–8 × 2.5–3.5 µm; conidia ellipsoidal, rough-walled, 3–3.5 × 2.5–3 µm.

*Typification*: CHINA. Hunan Province, Chenzhou City, Yizhang county, Mangshan National Nature Reserve, Jiangjunzhai, 24°59′7″N 112°52′24″E, soil, October 27 2015, Kai Chen, MS 18-1 (holotype HMAS 248819, ex-type strain CGMCC 3.18415).

*Notes*: In the phylogenetic tree (Fig. 1), *P. verrucisporum* appears as an independent terminal branch in subclade II. Among species of the subclade, *P. choerospondiatis* is similar in comparatively short stipes, but differs in no growth on CZ at 25 °C, less metulae per verticil, larger metulae and phialides, larger and finely roughened conidia, and slower growth on CYA, MEA and YES at 25 °C. The morphological differences among the related taxa are detailed in Table 1.

## Discussion

Members of *Penicillium* section *Sclerotiora* are characterized by the pigmented mycelia in shades of yellow and/or orange in culture, reverse view of colony yellow, orange or red, and sclerotia and cleistothecia if present bright-colored^12^. The conidiophores of the species in this section were mostly monoverticillate, and conidiophore branching pattern has not been considered of phylogenetic importance. In this work, we add three new species bearing biverticillate conidiophores to the section. Based on the four-gene phylogeny, three subclades in the section are recognized (Fig. 5). Species with monoverticillate conidiophores are in subclades I and III, while all those possessing biverticillate conidiophores are in subclade II. They join the species *P. herquei* and *P. malachiteum* recognized previously with a similar conidiophore branching pattern, which reveals that conidiophore branching pattern is phylogenetically informative. The result highlights that the morphological feature of conidiophore branching pattern is in accordant with the phylogenetic analysis and may be used as a reliable character in taxonomy of the section *Sclerotiora*.

Most species in *Penicillium* section *Sclerotiora* bearing monoverticillate conidiophores have vesiculate conidiophore apices, except for *P. adametzii*, *P. angulare* and *P. viticola* ^11, 35^. Although these three species do not form vesiculate conidiophores, they are scattered among or intertwined with those producing vesiculate conidiophores in the phylogenetic tree (Fig. 5). The former two species are located in subclade III and the latter is in subclade I. For the new species in subclade I, the conidiophores of *P. austrosinicum* and *P. exsudans* are vesiculate. The feature vesiculate conidiophore might not be phylogenetically informative for the group.

Sclerotia have been produced by seven members of the section *Sclerotia* ^28^. The color of sclerotia varies among species. For example, *P. austrosinicum* gives rise to cream to yellow sclerotia, and *P. hirayamae*, *P. sclerotiorum* and *P. vanoranjei* form orange ones on CYA at 25 °C^11,34^. In fact, sclerotia appear sporadically in *Penicillium* species across sections^34^, such as *P. corvianum* in section *Canescentia* producing brown sclerotia^26^, *P. macrosclerotiorum* in section *Gracilenta* giving rise to the white ones^36^, and *P. salamii* in section *Brevicompacta* having orange ones^21^. The production of sclerotia, as a morphological feature, does not reflect the phylogenetic relationships among species of the genus.

Species in section *Sclerotia* have been isolated from diverse substrates including soil, plants, and insects^28, 33, 37^. Apart from the species dominant in soil, the species collected from plant materials comprise a substantial proportion. Three of our five new species are from plant debris: *P. choerospondiatis* infecting the fruits of *Choerospondias axillaris*, and *P. austrosinicum* and *P. exsudans* from the rotten fruits of unidentified plants. Furthermore, *P. herquei* is on a leaf of *Agauria pyrifolia*, *P. hirayamae* is on cereals^11^, *P. viticola* infects grape^35^, and *P. cainii* has been isolated from nuts of *Juglans nigra* and *Carya ovata* ^34^. Some species are fungicolous: *P. angulare* is from a wood-decaying polypore^38^. Some species are living as plant endophytes^8–10^. Along with the identifications of new species, a broader range of substrates will be expected. *Penicillium herquei* can be planted by the nonsocial leaf-rolling weevil *Euops chinesis* ^39–41^, which indicates that the fungus evolved the ability to adapt divergent niches.

The genus *Penicillium* has been established for more than 200 years. New species of this genus have been increasingly found from different regions of the world, especially during the past two decades. The results of this study broaden our knowledge of the species diversity of this group. It is undoubted that more *Penicillium* species will be found in the unexplored areas of China as well as in other regions of the world based on the integrated or comprehensive studies of morphology, cultural characteristics and sequence data.

## Materials and Methods

### Fungal materials

Cultures were isolated and purified from the soil and rotten fruit samples collected in Guangdong, Hainan and Hunan provinces of China. Dried cultures have been deposited in the Herbarium Mycologicum Academiae Sinicae (HMAS), and the living ex-type strains are preserved in the China General Microbiological Culture Collection Center (CGMCC).

### Morphological observations

Morphological characterization of each sample was conducted following the standardized methods established by Visagie *et al*.^17^. Four standard growth media were used including the Czapek yeast autolysate agar (CYA, yeast extract Oxoid), malt extract agar (MEA, Amresco), yeast extract agar (YES) and Czapek’s agar (CZ). The methods for culture inoculation, incubation, microscopic examinations and digital recordings were described in our previous study^42^.

### DNA extraction, PCR amplification and sequencing

Fungal cultures were grown on the potato dextrose agar (PDA) medium for 7 d and then harvested for DNA extraction using the Plant Genomic DNA Kit (DP305, TIANGEN Biotech, Beijing, China). The fragments of the internal transcribed spacer region (ITS), beta-tubulin (*BenA*), calmodulin (*CaM*) and the second largest subunit of RNA polymerase II (*RPB2*) genes were amplified by PCR using the primers reported by Visagie *et al*.^17^. The products were purified and subject to sequencing on an ABI 3730 DNA Sequencer (Applied Biosystems).

### Phylogenetic analyses

The sequences obtained in this study have been deposited in GenBank. The accessions and those retrieved from GenBank^17^ are listed in Table 2. The sequences of each gene (i.e., ITS, *BenA*, *CaM* or *RPB2*) were aligned using the program MAFFT (ver. 7.221)^43^, and subsequently processed with BioEdit (ver. 7.1.10)^44^. The individual or concatenated gene data sets were used to generate the respective Maximum-Likelihood (ML) trees using the software MEGA (ver. 6.0.6)^45^ withthe most suitable nucleotide substitution model and 1,000 replicates of bootstrap tests. Bayesian Inference (BI) analysis was performed with MrBayes (ver. 3.2.5)^46^ using a Markov Chain Monte Carlo (MCMC) algorithm. Appropriate nucleotide substitution models and parameters were determined by using the program Modeltest (ver. 3.7)^47^. Four MCMC chains (one cold and three heated) were run for one million generations with the trees sampled every 100 generations. The first 25% trees were excluded as the burn-in phase of the analyses, and the posterior probability (PP) values were estimated with the 75% remaining trees. The consensus trees were viewed in FigTree (ver. 1.3.1; http://tree.bio.ed.ac.uk/software/figtree/). The species *Penicillium levitum* in section *Lanata-Divaricata* was used as an outgroup.

**Table 2:** Fungal species and sequences used in phylogenetic analyses.

Species	Collection	ITS	*BenA*	*CaM*	*RPB2*
*P. adametzii*	CBS 209.28 T^*^	JN714929	JN625957	KC773796	JN121455
*P. adametzioides*	CBS 313.59 T	JN686433	JN799642	JN686387	JN406578
*P. alexiae*	DTO118H8 T	KC790400	KC773778	KC773803	n.a.
*P. amaliae*	CV 1875 T	JX091443	JX091563	JX141557	n.a.
*P. angulare*	CBS 130293 T	AF125937	KC773779	KC773804	JN406554
*P. arianeae*	DTO20B8 T	KC773833	KC773784	KC773811	n.a.
*P. austrosinicum*	HMAS 248734 T	**KX885061** ^*^	**KX885041**	**KX885051**	**KX885032**
*P. bilaiae*	CBS 221.66 T	JN714937	JN625966	JN626009	JN406610
*P. brocae*	CBS 116113 T	AF484398	KC773787	KC773814	JN406639
*P. cainii*	DAOM 239914 T	JN686435	JN686366	JN686389	n.a.
*P. choerospondiatis*	HMAS 248813 T	**KX885063**	**KX885043**	**KX885053**	**KX885034**
	HMAS 248814	**KX885064**	**KX885044**	**KX885054**	**KX885035**
	HMAS 248815	**KX885065**	**KX885045**	**KX885055**	**KX885036**
*P. daejeonium*	CNU 100097 T	JX436489	JX436493	JX436491	n.a.
*P. exsudans*	HMAS 248735 T	**KX885062**	**KX885042**	**KX885052**	**KX885033**
*P. guanacastense*	DAOM 239912 T	JN626098	JN625967	JN626010	n.a.
*P. herquei*	CBS 336.48 T	JN626101	JN625970	JN626013	JN121494
	CBS 136.22	JN626100	JN625969	JN626012	n.a.
	CBS 347.51	JN626102	JN625971	JN626014	n.a.
	CBS 110644	JN626103	JN625972	JN626015	n.a.
*P*. cf. *herquei*	HMAS 248816	**KX885066**	**KX885046**	**KX885056**	**KX885037**
	HMAS 248817	**KX885067**	**KX885047**	**KX885057**	**KX885038**
	HMAS 248818	**KX885068**	**KX885048**	**KX885058**	**KX885039**
*P. hirayamae*	CBS 229.60 T	JN626095	JN625955	JN626003	JN121459
*P. jacksonii*	DAOM 239937 T	JN686437	JN686368	JN686391	n.a.
*P. johnkrugii*	DAOM 239943 T	JN686447	JN686378	JN686401	n.a.
*P. jugoslavicum*	CBS 192.87 T	KC773836	KC773789	KC773815	JN406618
*P. lilacinoechinulatum*	CBS 454.93 T	AY157489	KC773790	KC773816	n.a.
*P. malachiteum*	CBS 647.95 T	KC773838	KC773794	KC773820	n.a.
*P. mallochii*	DAOM 239917 T	JN626104	JN625973	JN626016	n.a.
*P. maximae*	NRRL 2060 T	EU427298	KC773795	KC773821	n.a.
*P. restingae*	MS-2014 T	KF803355	KF803349	KF803352	n.a.
*P. sanshaense*	HMAS 248820 T	**KX885070**	**KX885050**	**KX885060**	n.a.
*P. sclerotiorum*	CBS 287.36 T	JN626132	JN626001	JN626044	JN406585
*P. vanoranjei*	DTO99H6 T	KC695696	KC695686	KC695691	n.a.
*P. verrucisporum*	HMAS 248819 T	**KX885069**	**KX885049**	**KX885059**	**KX885040**
*P. viticola*	FKI-4410 T	AB606414	AB540174	n.a.	n.a.
*P. levitum*	CBS 345.48 T	GU981607	GU981654	KF296394	KF296432

T: ex-type strains.

n.a.: data not available.

^*^The accessions in bold are newly obtained in this study.
